# Medication Adherence and Risks of Mortality and End-Organ Damage in Asian Patients with Type 2 Diabetes: A Cohort Study from Southern Taiwan

**DOI:** 10.3390/biomedicines14030725

**Published:** 2026-03-22

**Authors:** Peng-Wen Chen, Ming-Chieh Lin, Tzu-Jung Fang, Mei-Yueh Lee

**Affiliations:** 1 Division of Endocrinology and Metabolism, Department of Internal Medicine, Kaohsiung Medical University Hospital, Kaohsiung 807377, Taiwan; abcdefg4004@gmail.com (P.-W.C.); u102001122@gap.kmu.edu.tw (M.-C.L.); tzujung66@gmail.com (T.-J.F.); 2 Division of Geriatrics and Gerontology, Department of Internal Medicine, Kaohsiung Medical University Hospital, Kaohsiung 807377, Taiwan; 3 School of Medicine, College of Medicine, Kaohsiung Medical University, Kaohsiung 807377, Taiwan; 4 Division of Endocrinology and Metabolism, Department of Internal Medicine, Kaohsiung Medical University Gangshan Hospital, Kaohsiung 820111, Taiwan

**Keywords:** type 2 diabetes, medication adherence, 8-item Morisky Medication Adherence Scale, all-cause mortality, cardiovascular outcomes, metabolic outcomes

## Abstract

**Background**: Medication adherence is a critical component of effective management in type 2 diabetes mellitus (T2DM). Although previous studies have explored the relationship between adherence and clinical outcomes, the strength and consistency of these associations have not been fully elucidated and remain unclear. In particular, evidence derived from patient-reported measures of adherence is limited, and the prognostic significance of adherence as assessed from the patient perspective is not clearly defined. **Methods**: We conducted a prospective observational cohort study consisting of adult patients with T2DM who received regular outpatient follow-up. Medication adherence was assessed at the time of enrollment using the eight-item Morisky Medication Adherence Scale (MMAS-8) and was categorized as good, moderate, or poor. Participants were subsequently followed for five years to ascertain clinical outcomes. Primary outcomes were assessed longitudinally and included the occurrence of nonfatal myocardial infarction, heart failure, nonfatal stroke, and progression to end-stage kidney disease (ESKD), as well as all-cause mortality. Secondary outcomes included changes in glycated hemoglobin (HbA1c), estimated glomerular filtration rate (eGFR), and low-density lipoprotein (LDL) levels. **Results**: No statistically significant differences were observed in the incidence of nonfatal myocardial infarction, heart failure, nonfatal stroke, or progression to ESKD across adherence groups. In contrast, all-cause mortality was significantly higher among patients with poor adherence. With respect to metabolic outcomes, HbA1c and eGFR at five years were comparable across adherence groups, whereas LDL levels were significantly higher in patients with poor adherence. **Conclusions**: Poor medication adherence as assessed at baseline may be related to a higher risk of all-cause mortality and poorer lipid control, while no statistically significant differences were observed for nonfatal cardiovascular or renal outcomes.

## 1. Introduction

Type 2 diabetes mellitus (T2DM) represents one of the most significant global health challenges, with its prevalence continuing to rise worldwide [1,2]. The growing burden of T2DM poses a substantial challenge to healthcare systems and is driven not only by the increasing number of affected individuals but also by the chronic and progressive nature of the disease [1]. T2DM is closely associated with a wide spectrum of long-term complications, including cardiovascular disease and end-stage kidney disease (ESKD), which together represent major contributors to morbidity, mortality, and healthcare expenditure [3,4,5,6,7]. Beyond these classical complications, accumulating evidence indicates that T2DM itself is independently associated with increased all-cause mortality, underscoring the systemic impact of the disease, which extends beyond vascular outcomes alone [3,8,9].

Although effective glycemic and metabolic control can mitigate many diabetes-related complications [3,7], a substantial proportion of patients fail to achieve the recommended treatment targets in real-world practice [10,11]. Medication adherence has been widely recognized as a critical determinant of therapeutic effectiveness in T2DM management [12]. Poor adherence to antidiabetic therapy has been associated with suboptimal glycemic control, increased hospitalization, higher healthcare costs, and an elevated risk of adverse clinical events [13,14,15,16,17,18]. Given the chronic and often asymptomatic nature of T2DM, sustained adherence to long-term pharmacotherapy is particularly challenging, and adherence behaviors may fluctuate over time.

While its clinical importance is recognized, the assessment of medication adherence remains challenging. Most prior studies have relied on pharmacy refill data or administrative claims to estimate adherence—data that primarily reflect medication availability rather than actual medication-taking behavior [13,14,15]. Such methods may fail to capture important patient-level factors, including beliefs about treatment, perceived barriers, treatment burdens, and day-to-day challenges encountered in routine clinical practice. Consequently, the relationship between medication adherence and long-term metabolic and clinical outcomes cannot be fully characterized when evaluated solely through objective utilization metrics.

In this context, patient-reported measures provide a complementary perspective by directly capturing adherence behaviors from the patient’s viewpoint. Standardized instruments such as the eight-item Morisky Medication Adherence Scale (MMAS-8) have been widely validated and applied across chronic diseases [19,20,21,22], offering a practical and structured approach to assessing real-world adherence. Patient-reported adherence may reflect broader aspects of health engagement, the capacity for self-management, and interactions with healthcare systems, which could have prognostic implications beyond glycemic control alone.

By integrating medication adherence as assessed through the MMAS-8 with both clinical endpoints and metabolic outcomes, the present study aims to provide a more comprehensive evaluation of the prognostic significance of medication adherence in patients with T2DM. Furthermore, given its focus on a predominantly Asian population from Southern Taiwan, this study adds to the existing literature on patient-reported adherence and long-term outcomes in this regional and ethnic context.

## 2. Materials and Methods

This study was a prospective observational cohort study conducted at the outpatient clinics of Kaohsiung Medical University Hospital, Taiwan, between January 2020 and December 2025. Adult patients aged over 20 years, who had a confirmed diagnosis of T2DM and received regular outpatient follow-up, were eligible for enrollment. At baseline, all participants completed a structured questionnaire—the Chinese version of the MMAS-8—to assess medication adherence. The MMAS-8 is a validated self-report instrument used to evaluate medication-taking behavior [23,24,25]. Scores range from 0 to 8, with higher scores indicating better adherence. Based on established criteria, participants were categorized into three adherence groups: good adherence (scores of 8), moderate adherence (scores of 6–7), and poor adherence (scores ≤ 5).

**Figure 1:**
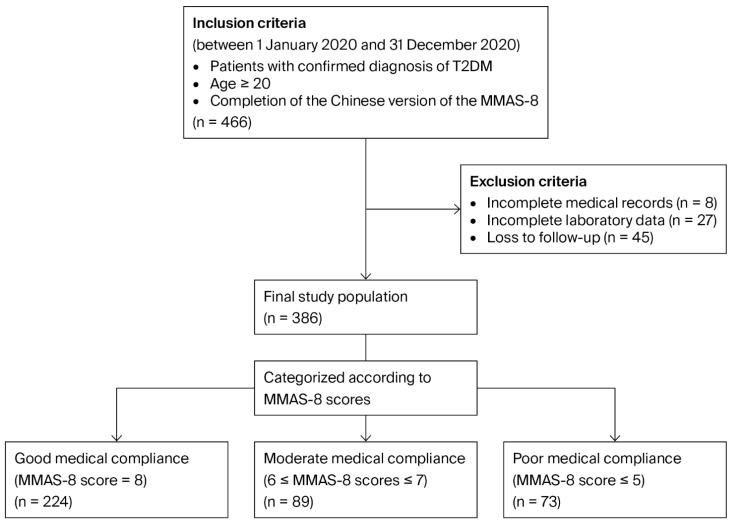
Flowchart of study population enrollment. Abbreviations: T2DM, type 2 diabetes mellitus; MMAS-8, 8-item Morisky Medication Adherence Scale.

After enrollment, participants were followed for five years to evaluate predefined clinical and metabolic outcomes. Patients with incomplete baseline clinical or laboratory data and those who were lost to follow-up during the study period were excluded from the final analysis. The study protocol was approved by the Institutional Review Board of Kaohsiung Medical University Hospital (KMUHIRB-E(I)-20190299), and all participants provided written informed consent prior to participation. Figure 1 shows the process of patient selection.

Antidiabetic treatment during follow-up was determined by treating physicians according to routine clinical practice. No protocol-mandated restrictions were placed on the choice of glucose-lowering medications, and treatment regimens could be adjusted at the clinicians’ discretion based on individual patient needs. However, detailed information regarding specific medication classes, treatment intensity, and dose adjustments during follow-up was not systematically recorded in the study dataset.

Baseline demographic characteristics, biochemical measurements, and comorbid conditions were obtained from medical records at enrollment. Primary endpoints were assessed longitudinally and included the occurrence of nonfatal myocardial infarction, heart failure, nonfatal stroke, and progression to ESKD, as well as all-cause mortality, during the follow-up period. Clinical events were identified through the review of medical records, and only the first occurrence of each event was included in the analysis. For the primary endpoint of all-cause mortality, power considerations were based on event-driven survival analysis. Sample size estimation was based on the detection of a clinically meaningful difference in 5-year all-cause mortality between groups using a Cox proportional hazards model, assuming a two-sided alpha of 0.05 and 80% power. Under these assumptions, we were able to detect hazard ratios of approximately 3.0 or greater between groups. Multivariable Cox models were used to estimate hazard ratios for the primary endpoint, adjusting for age, sex, HbA1c, LDL, hypertension, hyperlipidemia, CKD, prior MI, and heart failure. The proportional hazards assumption was assessed using Schoenfeld residuals for each covariate.

Secondary outcomes consisted of metabolic parameters, including the estimated glomerular filtration rate (eGFR), low-density lipoprotein (LDL) cholesterol levels, and changes in glycated hemoglobin (HbA1c) at 5 years. Blood tests for these metabolic parameters were conducted at baseline and at the end of the five-year follow-up period. During the follow-up period, additional laboratory measurements were conducted according to clinical indications and at the discretion of the treating physician; these were not uniformly scheduled across participants. Changes in metabolic parameters were evaluated by comparing the values measured at baseline with those obtained at the end of follow-up.

Categorical variables are presented as frequencies and percentages. Time-to-event analyses were performed to compare the incidence of clinical endpoints among the three study groups (good medical adherence, moderate adherence, and poor adherence groups). Cumulative incidence functions were estimated and compared using Gray’s test. For all-cause mortality, survival probabilities were estimated using the Kaplan–Meier method and compared using the log-rank test. Fine–Gray hazard models were applied for nonfatal outcomes to account for death as a competing risk, and Cox proportional hazards models were used for all-cause mortality. Statistical analysis was performed using IBM SPSS version 19.0.

## 3. Results

A total of 386 patients with T2DM were included in the final analysis. Among them, 224 patients (58.0%) were classified as having good adherence (MMAS-8 score of 8 points), 89 (23.1%) as having moderate adherence (6–7 points), and 73 (18.9%) as having poor adherence (≤5 points). Participants’ mean age was 64.16 years (good adherence group, 65.95 ± 9.61 years; moderate adherence group, 61.42 ± 11.83 years; poor adherence group, 62.02 ± 11.05 years). Baseline characteristics of the study population are summarized in Table 1.

The baseline body mass index (BMI) was 26.52 ± 3.79 kg/m^2^, 26.33 ± 4.36 kg/m^2^, and 27.22 ± 4.21 kg/m^2^ in the good adherence, moderate adherence, and poor adherence groups, respectively. Baseline HbA1c levels differed significantly across adherence groups and were highest in the poor adherence group (6.90 ± 0.97% in the good adherence group, 6.99 ± 0.96% in the moderate adherence group, and 7.32 ± 1.21% in the poor adherence group; *p* = 0.007). A stepwise increase in baseline LDL cholesterol levels was also observed across adherence categories, with the highest levels noted in the poor adherence group (72.86 ± 22.37 mg/dL in good adherence group, 77.80 ± 22.15 mg/dL in moderate adherence group, and 80.62 ± 22.28 mg/dL in poor adherence group; *p* = 0.020).

### 3.1. Primary Outcomes

No statistically significant differences were observed in the cumulative incidence of nonfatal myocardial infarction, heart failure, progression to ESKD, or nonfatal stroke across adherence groups during the five-year follow-up period (Table 2). In contrast, all-cause mortality was significantly higher in the poor adherence group (3.12% in the good adherence group, 8.98% in the moderate adherence group, and 15.06% in the poor adherence group, *p* = 0.001).

### 3.2. Secondary Outcomes

The evaluation of the secondary metabolic outcomes showed no significant differences regarding changes in HbA1c over the five-year period across adherence categories (0.19 ± 1.33% in good adherence group, −0.17 ± 1.42% in moderate adherence group, and 0.38 ± 1.81% in poor adherence group, *p* = 0.150) (Table 3). Renal function at five years, as assessed via eGFR, did not differ significantly from baseline; this was also comparable among groups. However, LDL cholesterol levels at the end of follow-up were significantly higher in patients with poor adherence than in those with good or moderate adherence (73.20 ± 22.46 mg/dL in good adherence group, 76.82 ± 22.64 mg/dL in moderate adherence group, and 85.18 ± 23.08 mg/dL in poor adherence group, *p* = 0.016).

## 4. Discussion

In this prospective cohort study, baseline medication adherence was not associated with a statistically significant difference in the risk of nonfatal myocardial infarction, stroke, heart failure, and progression to ESKD. However, patients with poor adherence at baseline exhibited a higher risk of all-cause mortality during follow-up. With respect to metabolic outcomes, changes in HbA1c levels and renal function did not differ substantially across adherence categories, whereas LDL cholesterol levels were higher among patients with poor adherence. These findings may suggest that poor adherence in patients with T2DM is more closely related to adverse overall survival and suboptimal lipid control than the occurrence of specific nonfatal cardiovascular or renal events.

Several investigations have suggested that poor adherence is associated with an increased risk of progression to ESKD. For example, Yaguchi et al. demonstrated that low adherence independently increased the risk of dialysis-dependent kidney failure [13], while Chang et al. found a higher risk of ESRD among patients with irregular use of oral antidiabetic agents [16]. In contrast, in our study, we did not observe a significant association between medication adherence and progression to ESKD.

While evidence regarding the association between medication adherence and cardiovascular outcomes is inconsistent, a relationship between medication adherence and all-cause mortality has been consistently demonstrated. Kim et al. found that lower adherence was associated with increased all-cause mortality and a higher incidence of cardiovascular disease overall, but not specifically with myocardial infarction [14]. Meanwhile, Khunti et al. reported that good adherence was associated with reduced risks of mortality and hospitalization rather than with individual cardiovascular events [15]. These discrepancies may be partly attributable to differences in study design and baseline kidney disease severity.

Considering the above, our findings align with previous evidence demonstrating that poor adherence is consistently associated with increased all-cause mortality. Among the 26 deaths observed in our cohort, the most common cause was malignancy (n = 11, 42.3%), followed by infection (n = 8, 30.8%). Detailed causes of death are presented in Appendix A Table A1. Epidemiological data further indicate that malignancy represents a leading cause of death among individuals with diabetes [26,27], indicating that mortality in this population is driven not only by vascular complications but also by other factors, including infection and acute illness [28,29,30]. Under these circumstances, all-cause mortality may serve as a more sensitive and earlier indicator of inadequate overall health management than specific nonfatal clinical endpoints. It is possible that medication adherence reflects broader aspects of overall chronic disease management rather than the isolated effects of glucose-lowering therapy. Patients with poor adherence may be less engaged in their overall healthcare, which encompasses lifestyle behaviors, follow-up attendance, and adherence to concomitant therapies for comorbid conditions. Poor medication adherence may thus function as a surrogate marker of reduced healthcare engagement, leading to the delayed recognition of acute illness and suboptimal management of nondiabetic conditions. However, this interpretation should be considered hypothesis-generating, as important factors such as lifestyle behaviors, healthcare access, psychosocial factors, education, and mental health were not directly measured in the present study. Future studies incorporating these variables may help clarify the mechanisms underlying the observed associations.

Baseline HbA1c levels were already higher in the poor adherence group compared with the other adherence categories. However, the changes in HbA1c over the five-year follow-up period did not differ significantly across groups. Because our analysis did not include strict adjustment for multiple behavioral and clinical factors that may influence glycemic control, these findings should be interpreted cautiously. Nevertheless, the observed pattern may suggest that individuals with poorer medication adherence tend to have less optimal glycemic control. At the same time, glycemic outcomes in patients with T2DM are influenced by multiple factors beyond pharmacotherapy alone, including dietary patterns, physical activity, insulin titration practices, and structured care programs such as shared care initiatives for diabetes, which are commonly implemented in Taiwan. These systemic and behavioral influences may attenuate the measurable impact of medication adherence on glycemic outcomes [31,32,33].

In contrast, the association between poor adherence and elevated LDL cholesterol levels highlights the broader metabolic consequences of adherence behaviors. Notably, LDL cholesterol levels tended to be higher in patients with poorer adherence both at baseline and at the end of the five-year follow-up, suggesting a consistent pattern of less optimal lipid control among individuals with lower adherence. Suboptimal adherence to antidiabetic therapy may also reflect more generalized nonadherence to other chronic medications, including lipid-lowering agents, or reduced compliance with dietary recommendations, which may contribute to inadequate lipid control. Although we did not observe a significant association between medication adherence and nonfatal myocardial infarction, the control of metabolic risk factors remains important in shaping cardiovascular outcomes among patients with T2DM [34,35].

Several limitations of this study should be acknowledged. First, self-reported adherence is subject to recall and social desirability bias, and the follow-up duration and event rates may have limited the ability to detect differences in outcomes characterized by slow development, such as ESKD and nonfatal cardiovascular events. Second, medication adherence was assessed only once at baseline and may have changed during the follow-up period. Because adherence behavior is dynamic, a single baseline measurement may not fully capture long-term adherence patterns. Therefore, the present findings should be interpreted as reflecting the prognostic significance of baseline adherence status, rather than the sustained effects of adherence over time. Moreover, reverse causality between metabolic control and adherence behaviors could not be fully excluded in this observational study. Third, detailed information on specific medication classes, treatment intensity, and dose adjustments during follow-up was not systematically available in our dataset. Therefore, we were unable to adjust for differences in pharmacotherapy patterns across adherence groups. Because medication regimens may influence outcomes such as mortality, lipid control, glycemic changes, and renal function, residual confounding related to pharmacologic treatment cannot be excluded. Fourth, as this was a single-center study, its generalizability may be limited. Fifth, the number of events for several clinical endpoints was relatively small. Consequently, the study may have been underpowered to detect modest associations between medication adherence and these outcomes. Therefore, the absence of statistically significant differences should not be interpreted as evidence of no association, and these findings should be considered exploratory. Sixth, the exclusion of patients lost to follow-up may have introduced selection bias, and residual attrition bias could not be entirely excluded. Seventh, outcomes were determined through medical record review; therefore, some degree of misclassification was possible. Lastly, residual confounding due to unmeasured factors, including lifestyle, socioeconomic, and psychological variables, could not be completely excluded.

Future studies integrating patient-reported adherence measures with objective data, such as those derived from pharmacy refill records or electronic monitoring systems, may facilitate a more complete assessment of adherence behaviors. Larger cohort studies with longer follow-up periods, incorporating repeated or time-varying adherence assessments and interventional designs, are also warranted to determine whether targeted strategies to improve adherence can facilitate meaningful reductions in mortality.

In conclusion, while poor medication adherence at baseline in patients with T2DM was not significantly associated with increased risks of nonfatal myocardial infarction, stroke, heart failure, or ESKD, it may be related to a higher risk of all-cause mortality and poorer lipid control.

## Figures and Tables

**Table 1:** Demographics of enrolled patients.

	Good Medication Adherence	Moderate Medication Adherence	Poor Medication Adherence	Total Patients	*p* Value
Total, n	224	89	73	386	
Age (years)	65.95 ± 9.61	61.42 ± 11.83	62.02 ± 11.05	64.16 ± 10.62	<0.001
Male sex, n (%)	133 (59.4%)	48 (53.9%)	39 (53.4%)	220 (57.0%)	
Biochemical Data					
BMI (kg/m^2^)	26.52 ± 3.79	26.33 ± 4.36	27.22 ± 4.21	26.61 ± 4.01	0.326
FPG (mg/dL)	134.19 ± 38.17	129.72 ± 31.96	136.03 ± 30.40	133.50 ± 35.41	0.481
HbA1c (%)	6.90 ± 0.97	6.99 ± 0.96	7.32 ± 1.21	7.01 ± 1.03	0.007
ALT (U/mL)	27.13 ± 14.23	27.16 ± 15.67	25.79 ± 14.19	26.88 ± 14.54	0.779
Total cholesterol (mg/dL)	145.92 ± 29.25	167.06 ± 29.55	157.47 ± 30.69	152.41 ± 67.69	0.043
TG (mg/dL)	107.25 ± 58.75	114.73 ± 74.26	119.84 ± 79.03	111.35 ± 66.74	0.325
HDL (mg/dL)	46.98 ± 12.86	48.08 ± 15.26	46.76 ± 13.16	47.19 ± 13.47	0.776
LDL (mg/dL)	72.86 ± 22.37	77.80 ± 22.15	80.62 ± 22.28	75.47 ± 22.47	0.020
eGFR (mL/min/1.73 m^2^)	74.41 ± 23.76	79.70 ± 24.51	79.18 ± 24.47	76.53 ± 24.14	0.126
UACR (mg/g)	126.29 ± 491.99	78.57 ± 172.52	190.44 ± 612.00	127.47 ± 467.02	0.318
Underlying Diseases, n (%)					
HTN	155 (69.2%)	60 (67.4%)	46 (63.0%)	261 (67.6%)	
Hyperlipidemia	191 (85.3%)	75 (84.3%)	62 (84.9%)	328 (85.0%)	
CKD	59 (26.3%)	26 (29.2%)	15 (20.5%)	100 (25.9%)	

BMI, body mass index; FPG, fasting plasma glucose; HbA1c, glycated hemoglobin; ALT, alanine aminotransferase; TG, triglycerides; HDL, high-density lipoprotein; LDL, low-density lipoprotein; eGFR, estimated glomerular filtration rate; UACR, urine albumin–creatinine ratio; HTN, hypertension; CKD, chronic kidney disease.

**Table 2:** Primary endpoint results.

	Good Medication Adherence	Moderate Medication Adherence	Poor Medication Adherence	*p* Value
Accumulative Event at 5 Years, n (%)				
Nonfatal MI	12 (6.69%)	2 (2.24%)	5 (6.84%)	0.351
Heart Failure	17 (7.58%)	5 (5.61%)	5 (6.84%)	0.856
Renal Failure	3 (1.33%)	0 (0%)	1 (1.36%)	0.522
Nonfatal Stroke	10 (4.46%)	4 (4.49%)	1 (1.36%)	0.484
Death	7 (3.12%)	8 (8.98%)	11 (15.06%)	0.001 *

MI, myocardial infarction; * indicates statistical significance.

**Table 3:** Secondary outcomes.

	Good Medication Adherence	Moderate Medication Adherence	Poor Medication Adherence	*p* Value
Change at 5 years				
HbA1c (%)	0.19 ± 1.33	−0.17 ± 1.42	0.38 ± 1.81	0.150
Data at 5 years				
eGFR (mL/min/1.73 m^2^)	78.23 ± 24.46	84.30 ± 24.09	76.11 ± 26.79	0.210
LDL (mg/dL)	73.20 ± 22.46	76.82 ± 22.64	85.18 ± 23.08	0.016 *

HbA1c, glycated hemoglobin; eGFR, estimated glomerular filtration rate; LDL, low-density lipoprotein. * indicates statistical significance.

## Data Availability

The data presented in this study are available on request from the corresponding author. The data are not publicly available due to privacy restrictions.

## Abbreviations

The following abbreviations are used in this manuscript:

T2DM	Type 2 diabetes mellitus
MMAS-8	8-Item Morisky Medication Adherence Scale
ESKD	End-stage kidney disease
HbA1c	Glycated hemoglobin
eGFR	Estimated glomerular filtration rate
LDL	Low-density lipoprotein
HR	Hazard ratio
CI	Confidence interval
BMI	Body mass index

## Appendix A

**Table A1:** Causes of death.

Total (n = 26)	n
Malignancy (n = 11, 42.3%)	
Lung cancer	4
Hepatocellular carcinoma	3
Diffuse large B-cell lymphoma	1
Thymic squamous cell carcinoma	1
Cholangiocarcinoma	1
Gastric adenocarcinoma	1
Cardiovascular events (n = 2, 7.7%)	
Acute ischemic stroke	1
Acute myocardial infarction	1
Infection (n = 8, 30.8%)	
Pneumonia	3
Urinary tract infection	2
COVID-19	2
Liver abscess	1
Others (n = 5, 19.2%)	
Major trauma	2
Peptic ulcer perforation	1
Abdominal aortic aneurysm rupture	1
Acute pancreatitis	1
